# 单倍体造血干细胞移植后EB病毒激活患者早期细胞免疫功能耗竭的研究

**DOI:** 10.3760/cma.j.cn121090-20240825-00322

**Published:** 2024-11

**Authors:** 怡霏 黄, 珊毓 张, 家宝 何, 雅 周, 容涛 薛, 志平 范, 芬 黄, 娜 许, 竞 孙, 启发 刘, 韧 林

**Affiliations:** 南方医科大学南方医院血液内科，广东省血液病临床研究中心，广州 510515 Department of Hematology, Nanfang Hospital, Southern Medical University, Clinical Medical Research Center of Hematological Diseases of Guangdong Province, Guangzhou 510515, China

**Keywords:** 异基因造血干细胞移植, EB病毒感染, 免疫重建, 耗竭标志物, 免疫功能, Allogeneic hematopoietic stem cell transplantation, Epstein-barr virus, Immune reconstitution, Exhaustion markers, Immune function

## Abstract

**目的:**

本研究通过检测单倍体造血干细胞移植（HSCT）后早期外周血NK细胞、T细胞和B细胞亚群及功能变化情况，探讨移植早期免疫重建与EB病毒（EBV）激活的关联。

**方法:**

纳入23例接受单倍体HSCT患者，比较移植后发生EBV激活（EBV+组）与未激活（EBV−组）患者移植后1、2、3个月的NK细胞、T细胞和B细胞免疫重建情况以及NK细胞和T细胞耗竭标志物（PD-1、TIM-3、CTLA-4）的表达水平及其杀伤功能的差异。

**结果:**

EBV+组9例，均为EBV血症，未发生EBV相关终末器官疾病；EBV−组14例。两组患者基线临床特征差异均无统计学意义。移植后1个月EBV+组CD3^+^CD8^+^ T细胞中位比例低于EBV−组（*P*＝0.033）；移植后2个月EBV+组患者CD3^-^CD16^neg^CD56^bri^ NK细胞亚群中位比例高于EBV−组（*P*＝0.046）；移植后1、2、3个月两组间CD3^-^CD19^+^ B细胞中位比例差异均无统计学意义。移植后1个月EBV+组CD3^-^CD16^bri^CD56^dim^ NK细胞亚群上CTLA-4的表达水平高于EBV−组（*P*＝0.033），EBV+组CD3^+^CD8^+^ T细胞上TIM-3的表达水平高于EBV−组（*P*＝0.009）；移植2个月EBV+组CD3^-^CD16^neg^CD56^dim^ NK细胞亚群上TIM-3的表达水平低于EBV−组（*P*＝0.023）；移植后1、3个月EBV+组CD3^+^CD4^+^ T细胞上TIM-3的表达水平均高于EBV−组（*P*＝0.002、*P*＝0.043）。移植后1个月EBV+组CD3^+^CD8^+^ T和CD3^+^CD4^+^ T细胞表达颗粒酶B的中位阳性率低于EBV−组（*P*＝0.033、*P*＝0.016）；移植后2个月EBV+组CD3^-^CD16^bri^CD56^neg^细胞亚群表达颗粒酶B的中位阳性率高于EBV−组（*P*＝0.012）；EBV+组CD3^+^CD4^+^ T细胞表达颗粒酶B中位阳性率低于EBV−组（*P*＝0.049）；移植后3个月EBV+组CD3^-^CD16^bri^CD56^dim^细胞亚群表达穿孔素的中位阳性率高于EBV−组（*P*＝0.003）；EBV+组CD3^+^CD8^+^ T细胞表达IFN-γ中位阳性率低于EBV−组（*P*＝0.036）。

**结论:**

单倍体HSCT后NK细胞与T淋巴细胞重建延迟、耗竭分子高表达及杀伤功能减弱可能与移植后EBV激活相关。

## Abstract (en)

**Objective:**

This study aimed to investigate the association between early immune reconstitution and Epstein-Barr virus（EBV）reactivation by analyzing changes in natural killer（NK）, B, and T cells and their functional status in the peripheral blood during the early post-transplant period.

**Methods:**

This study included 23 patients who underwent haplo-hematopoietic stem cell transplantation（HSCT）. The immune reconstitution of NK cells, T cells, and B cells as well as the expression levels of NK and T cell exhaustion markers（PD-1, TIM-3, and CTLA-4）and cytotoxic function at 1, 2, and 3 months post-transplantation were compared between patients with EBV activation（EBV+ group）and those without activation（EBV− group）post-transplantation.

**Results:**

EBV activation occurred in nine patients post-transplantation（EBV+ group）, whereas 14 patients demonstrated no activation（EBV− group）. All patients with EBV activation exhibited EBV viremia, and no EBV-associated diseases occurred. No significant differences in the clinical characteristics were found between the two groups of patients. The median proportion of CD3^+^CD8^+^ T cells in the EBV+ group was significantly lower than that in the EBV− group at 1 month post-transplantation（*P*＝0.033）. The median proportion of the CD3^-^CD16^neg^CD56^bri^ subset in the EBV+ group was significantly higher than that in the EBV− group at 2 months post-transplantation（*P*＝0.046）. No significant differences in the median proportions of CD3^-^CD19^+^ B cells were observed between the two groups at 1, 2, and 3 months post-transplantation. The expression of CTLA-4 on CD3^-^CD16^bri^CD56^dim^ NK cells in the EBV+ group was significantly higher than that in the EBV− group at 1 month post-transplantation（*P*＝0.033）. The expression of TIM-3 on CD3^+^CD8^+^ T cells in the EBV+ group was significantly higher than that in the EBV− group（*P*＝0.009）. The expression level of TIM-3 on CD3^-^CD16^neg^CD56^dim^ NK cells in the EBV+ group was significantly lower than that in the EBV− group at 2 months post-transplantation（*P*＝0.023）. The expression levels of TIM-3 on CD3^+^CD4^+^ T cells in the EBV+ group than those in the EBV− group at 1 and 3 months post-transplantation（*P*＝0.002, *P*＝0.043）. The median positive rate of Granzyme B expression in CD3^+^CD8^+^ T cells and CD3^+^CD4^+^ T cells in the EBV+ group was significantly lower than that in the EBV− group at 1-month post-transplantation（*P*＝0.033, *P*＝0.016）. The median positive rate of Granzyme B expression in the CD3^-^CD16^bri^CD56^neg^ cell subset in the EBV+ group was higher than that in the EBV− group at 2 months post-transplantation（*P*＝0.012）. The median positive rate of Granzyme B expression in CD3^+^CD4^+^ T cells in the EBV+ group remained significantly lower than that in the EBV− group at 2 months post-transplantation（*P*＝0.049）. The median positive rate of perforin expression in the CD3^-^CD16^bri^CD56^dim^ cell subset was significantly higher in the EBV+ group than in the EBV− group at 3 months post-transplantation（*P*＝0.003）. The median positive rate of IFN-γ expression in CD3^+^CD8^+^ T cells in the EBV+ group was significantly lower than that in the EBV− group at 3 months post-transplantation（*P*＝0.036）.

**Conclusion:**

Delayed NK cell and T lymphocyte reconstitution, high exhaustion marker expression, and weakened cytotoxic functions may be related to EBV reactivation after haploidentical HSCT.

异基因造血干细胞移植（allo-HSCT）是治疗多种血液系统恶性和非恶性疾病的重要手段。而移植后机会性感染，尤其是移植后病毒感染，是导致预后不良的重要并发症[1]。移植后Epstein-Barr病毒（EBV）激活的患者可发生包括致死性移植后淋巴细胞增殖性疾病（post-transplant lymphoproliferative disease, PTLD）在内的相关终末器官疾病。随着单倍体HSCT在临床中的广泛应用，文献报道allo-HSCT后EBV激活的发生率可高达65％，EBV相关PTLD（EBV-PTLD）的发病率可高达26％，影响患者长期生存[2]–[3]。

EBV感染及相关疾病的发生和发展与患者的免疫状态密切相关。EBV主要感染B细胞和上皮细胞[4]，在病毒感染尤其是长期潜伏慢性病毒感染患者中存在T细胞和NK细胞耗竭，主要表现为T细胞和NK细胞增殖减低、凋亡/程序死亡增加、多种耗竭标志物（如PD-1、TIM-3、CTLA-4及2B4等）高表达、杀伤能力和细胞因子分泌减低等[5]–[7]，是病毒免疫逃逸的主要机制之一[8]。研究显示在EBV相关性疾病如鼻咽癌、EBV相关淋巴瘤及慢性活动性EBV感染中存在T细胞和NK细胞耗竭[9]–[10]。但单倍体HSCT患者移植后EBV激活是否与T细胞、NK细胞耗竭相关，报道较少。在本研究中，我们探讨了单倍体HSCT患者移植早期T细胞和NK细胞免疫重建及耗竭标志物表达情况与移植后EBV激活的相关性。

## 病例与方法

1. 病例：回顾性分析2023年11月至2024年2月在南方医科大学南方医院血液内科连续接受单倍体HSCT的血液病患者资料，纳入23例具有完整移植早期（移植后1、2、3个月）免疫重建数据的患者。

2. 预处理及移植物抗宿主病（GVHD）预防方案：预处理方案包括白消安（Bu）联合环磷酰胺（CTX）、Bu联合氟达拉滨（Flu）及全身照射（TBI）联合CTX和依托泊苷[11]。GVHD的预防均采用短期甲氨蝶呤、环孢素A、吗替麦考酚酯联合抗人胸腺细胞免疫球蛋白（ATG）方案。

3. EBV血症监测：移植后3个月内每周采用实时荧光定量PCR监测外周血EBV-DNA水平。一旦血液中的EBV-DNA呈阳性，次日再次检测病毒载量。EBV血症定义为血浆中EBV-DNA连续两次>500拷贝数/ml[12]。

4. 流式细胞术鉴定NK细胞、T细胞及B细胞亚群以及功能改变：通过Ficoll密度梯度离心分离外周血单个核细胞（PBMC），进行CD3^+^、CD56^+^、CD16^+^、CD4^+^、CD8^+^、CD19^+^抗体染色，将NK细胞分为CD3^-^CD16^neg^ CD56^dim^、CD3^-^CD16^neg^CD56^bri^、CD3^-^ CD16^bri^ CD56^neg^、CD3^-^CD16^bri^CD56^dim^四个亚群，将T细胞分为CD3^+^CD4^+^、CD3^+^CD8^+^两个亚群，CD3^-^CD19^+^标记B细胞。通过表面染色检测T细胞及NK细胞耗竭标志物PD-1、TIM-3和CTLA-4的表达水平。为了对CTLA-4进行最大程度（表面和细胞内）染色，在表面染色后固定并透化细胞，与CTLA-4或同型对照抗体在室温下继续孵育30 min。使用佛波酯（50 ng/ml）和离子霉素（1 µg/ml）非特异性刺激T细胞，同时通过佛波酯、离子霉素及K562细胞系刺激NK细胞，诱导其分泌胞内因子，细胞透化后检测IFN-γ、TNF-α、颗粒酶B和穿孔素水平以反映细胞功能。应用FACS CANTO Ⅱ型流式细胞仪（美国BD公司）进行流式细胞分析，FlowJo 10.6分析流式细胞术数据。

5. 统计学处理：运用GraphPad Prism 6和SPSS 26.0软件进行统计分析。对于分类数据，采用卡方检验或Fisher精确检验；对于计量数据，采用Mann-Whitney *U*检验或Kruskal-Wallis *H*检验。双侧*P*<0.05为差异有统计学意义。

## 结果

1. 患者基本特征：患者的中位年龄为38（15～64）岁，其中男16例（69.6％），女7例（30.4％）。移植后100 d内9例患者发生EBV激活（EBV+组），全部为EBV血症，14例未发生EBV激活（EBV−组），两组患者基线临床特征差异均无统计学意义（表1）。

**表1:** 单倍体造血干细胞移植后EBV激活（EBV+）与未激活（EBV−）组患者基线临床特征比较

临床特征	EBV+组（9例）	EBV−组（14例）	*P*值
发病年龄［岁，*M*（范围）］	34（20～64）	40（15～58）	0.439
性别［例（％）］			0.675
男	7（77.8）	9（64.3）	
女	2（22.2）	5（35.7）	
疾病诊断［例（％）］			0.226
急性髓系白血病	7（77.8）	5（35.7）	
急性淋巴细胞白血病	2（22.2）	6（42.9）	
骨髓增生异常综合征	0（0）	2（14.3）	
骨髓纤维化	0（0）	1（7.1）	
移植物来源［例（％）］			0.336
外周血+骨髓	3（33.3）	7（50.0）	
外周血+骨髓+脐血	1（11.1）	4（28.6）	
外周血+脐血	5（55.6）	3（21.4）	
预处理方案［例（％）］			0.400
Bu为主	6（66.7）	8（57.1）	
TBI为主	3（33.3）	6（42.9）	
HLA配型［例（％）］			1.000
5/10	6（66.7）	8（57.1）	
>5/10	3（33.3）	6（42.9）	
移植前疾病状态［例（％）］			1.000
CR	9（100.0）	13（92.9）	
非CR	0（0）	1（7.1）	
供者年龄［岁，*M*（范围）］	28（9～40）	33（13～60）	0.141
供者性别［例（％）］			1.000
男	6（66.7）	10（71.4）	
女	3（33.3）	4（28.6）	
回输MNC计数［×10^8^/kg，*M*（范围）］	11.1（10.1～25.7）	11.0（8.7～18.0）	0.277

**注** Bu：白消安；TBI：全身照射；HLA：人类白细胞抗原；CR：完全缓解；MNC：单个核细胞

2. EBV血症和EBV相关疾病情况：9例患者发生EBV激活，中位发生时间为+45（+14～+81）d，均为EBV血症，病毒载量峰值中位数为4 760（540～17 800）拷贝数/ml。9例患者均减量免疫抑制剂，其中4例患者接受利妥昔单抗治疗，1例接受EBV特异性细胞毒性T淋巴细胞（EBV-CTL）治疗。8例患者外周血EBV-DNA转阴，中位转阴时间为11.5（4～20）d；1例患者持续未转阴，最终于+98 d因肠道急性GVHD死亡。无患者进展为EBV相关疾病。

3. NK细胞、T细胞及B细胞免疫重建：移植后2个月，EBV+组患者CD16^neg^CD56^bri^亚群中位比例高于EBV−组［0.80％（0～11.00％）对0.03％（0～7.48％），*P*＝0.046］，两组间其他NK细胞亚群中位比例差异均无统计学意义（图1A～D）。移植后1个月，EBV+组CD3^+^CD8^+^ T细胞中位比例低于EBV−组［9.17％（0.27％～20.40％）对20.35％（2.31％～71.5％），*P*＝0.033］，移植后2、3个月，两组的CD3^+^CD8^+^ T细胞中位比例差异无统计学意义（图1E）。移植后1、2、3个月，两组间CD3^+^CD4^+^ T细胞中位比例差异均无统计学意义（图1F）。移植后1、2、3个月，两组间CD3^-^CD19^+^ B细胞中位比例差异均无统计学意义（图1G）。

**图1:**
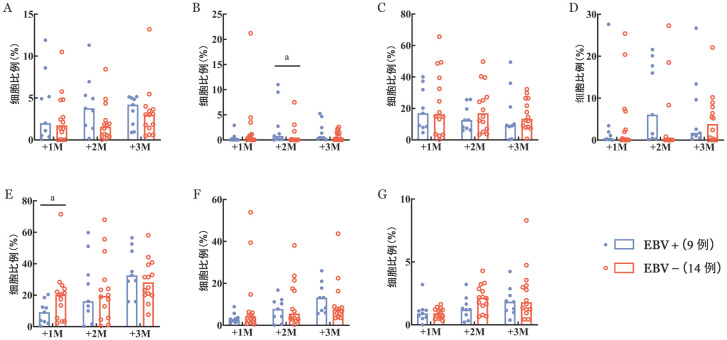
NK细胞、T细胞及B细胞免疫重建情况（^a^*P*<0.05） **A** CD16^neg^CD56^dim^ NK细胞比例；**B** CD16^neg^CD56^bri^ NK细胞比例；**C** CD16^bri^CD56^neg^ NK细胞比例；**D** CD1^bri^CD56^dim^ NK细胞比例；**E** CD3^+^CD8^+^ T细胞比例；**F** CD3^+^CD4^+^ T细胞比例；**G** CD19^+^ B细胞比例 **注** +1M：移植后1个月；+2M：移植后2个月；+3M：移植后3个月

4. NK细胞耗竭标志物检测：移植后1、2、3个月，两组间NK细胞亚群上耗竭标志物（PD-1、TIM-3、CTLA-4）的表达水平如图2所示。移植后1个月，EBV+组中CD16^bri^CD56^dim^亚群上CTLA-4的表达水平高于EBV−组［21.2％（0～65.2％）对1.14％（0～62.5％），*P*＝0.033］，移植后2、3个月时两组间差异无统计学意义。移植后1、2、3个月，两组间其他NK细胞亚群上CTLA-4的表达水平差异均无统计学意义。移植后2个月，EBV+组中CD16^neg^CD56^dim^亚群上TIM-3的表达水平低于EBV−组［5.56％（0～21.2％）对10.0％（1.8％～34.0％），*P*＝0.023］，移植后1、3个月时两组间差异均无统计学意义。移植后1、2、3个月，两组间CD16^bri^CD56^dim^、CD16^bri^CD56^neg^、CD16^neg^CD56^bri^细胞亚群上TIM-3的表达水平差异均无统计学意义。移植后1、2、3个月，两组间各NK细胞亚群上PD-1的表达水平差异均无统计学意义。

**图2:**
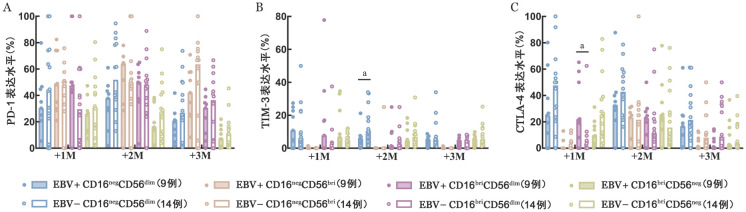
NK细胞耗竭标志物检测结果（^a^*P*<0.05） **A** PD-1表达水平；**B** TIM-3表达水平；**C** CTLA-4表达水平 **注** +1M：移植后1个月；+2M：移植后2个月；+3M：移植后3个月

5. T细胞耗竭标志物检测：移植后1、2、3个月，两组间CD3^+^CD8^+^和CD3^+^CD4^+^ T细胞上耗竭标志物（PD-1、TIM-3、CTLA-4）的表达水平如图3所示。移植后1个月，EBV+组中CD3^+^CD8^+^ T细胞上TIM-3的表达水平高于EBV−组［4.60％（0.24％～24.00％）对1.09％（0.21％～5.16％），*P*＝0.009］，移植后2、3个月时两组间差异无统计学意义。移植后1、3个月，两组患者CD3^+^CD4^+^ T细胞上TIM-3表达水平均高于EBV−组［16.30％（1.15％～57.10％）对1.99％（0～35.00％），*P*＝0.002；4.80％（2.60％～13.50％）对2.46％（0～6.74％），*P*＝0.043］，移植后2个月两组间差异均无统计学意义。移植后1、2、3个月，CD3^+^CD8^+^和CD3^+^CD4^+^ T细胞上PD-1和CTLA-4表达水平差异均无统计学意义。

**图3:**
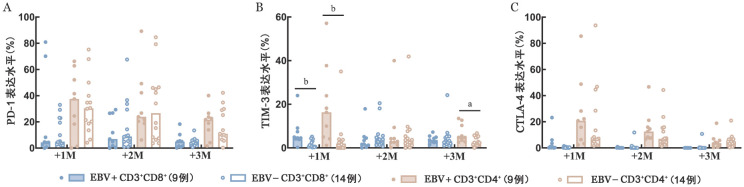
T细胞耗竭标志物检测结果（^a^*P*<0.05、^b^*P*<0.01） **A** PD-1表达水平；**B** TIM-3表达水平；**C** CTLA-4表达水平 **注** +1M：移植后1个月；+2M：移植后2个月；+3M：移植后3个月

6. NK细胞功能检测：移植后2个月，EBV+组CD16^bri^CD56^neg^细胞亚群表达颗粒酶B的中位阳性率高于EBV−组［24.75％（4.98％～77.10％）对3.58％（0.36％～96.80％），*P*＝0.012］，移植后1、3个月两组间差异均无统计学意义。移植后3个月，CD16^bri^CD56^dim^细胞亚群表达穿孔素的中位阳性率高于EBV−组［87.95％（3.20％～98.13％）对50.00％（0.20％～89.80％），*P*＝0.003］，移植后1、2个月两组间差异均无统计学意义。移植后1、2、3个月，两组间CD16^bri^CD56^neg^细胞亚群表达穿孔素和CD16^bri^CD56^dim^细胞亚群表达颗粒酶B的中位阳性率以及两组间CD16^neg^CD56^bri^和CD16^neg^CD56^dim^细胞亚群表达TNF-α、IFN-γ的中位阳性率差异均无统计学意义（图4）。

**图4:**
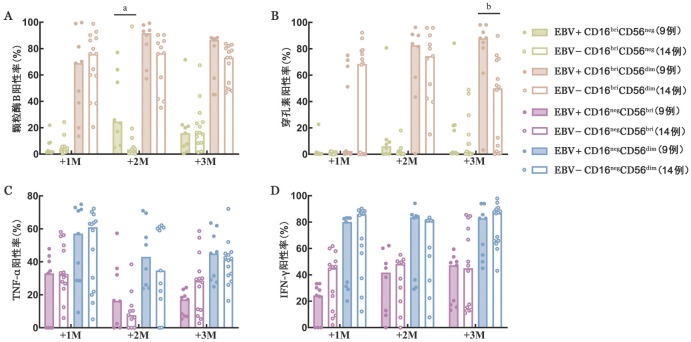
NK细胞功能检测结果（^a^*P*<0.05、^b^*P*<0.01） **A** 颗粒酶B阳性率；**B** 穿孔素阳性率；**C** TNF-α阳性率；**D** IFN-γ阳性率 **注** +1M：移植后1个月；+2M：移植后2个月；+3M：移植后3个月

7. T细胞功能检测：移植后1个月，EBV+组CD3^+^CD8^+^ T细胞表达颗粒酶B的中位阳性率低于EBV−组［43.00％（0.18％～82.60％）对74.60％（23.10％～98.50％），*P*＝0.033］，移植后2、3个月时两组间差异均无统计学意义。移植后3个月，EBV+组CD3^+^CD8^+^ T细胞表达IFN-γ的中位阳性率低于EBV−组［17.70％（4.40％～36.90％）对34.30％（15.70％～44.95％），*P*＝0.036］，移植后1、2个月两组间差异无统计学意义。移植后1、2、3个月，两组间CD3^+^CD8^+^ T细胞表达穿孔素和TNF-α的中位阳性率差异无统计学意义。移植后1、2个月，EBV+组CD3^+^CD4^+^ T细胞表达颗粒酶B中位阳性率均低于EBV−组［6.68％（2.54％～24.00％）对22.55％（0.76％～67.20％），*P*＝0.016；11.70％（0.27％～23.20％）对24.90％（4.16％～91.34％），*P*＝0.049］，移植后3个月时两组间差异均无统计学意义。移植后1、2、3个月，两组间CD3^+^CD4^+^ T细胞表达穿孔素、IFN-γ、TNF-α的中位阳性率差异均无统计学意义（图5）。

**图5:**
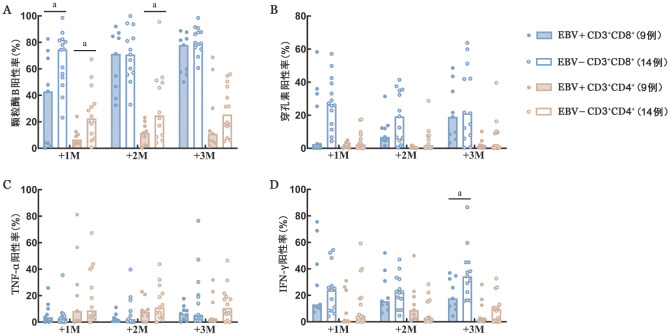
T细胞功能检测结果（^a^*P*<0.05） **A** 颗粒酶B阳性率；**B** 穿孔素阳性率；**C** TNF-α阳性率；**D** IFN-γ阳性率 **注** +1M：移植后1个月；+2M：移植后2个月；+3M：移植后3个月

## 讨论

移植后T细胞和NK细胞的免疫重建对控制包括EBV在内的潜伏病毒激活至关重要[13]–[14]。本研究分析了23例接受单倍体HSCT的患者移植后第1、2、3个月的NK细胞、T细胞、B细胞重建及细胞免疫功能的动态变化。结果显示，移植后发生EBV激活的患者存在移植早期NK细胞和T淋巴细胞免疫重建延迟、耗竭标志物表达增高及杀伤功能减弱。

NK细胞是移植后首先重建的细胞亚群，其数量在移植后1～2个月内恢复正常[15]，因此NK细胞在移植后早期抗病毒免疫中具有重要作用，是抵御包括EBV在内多种病毒的第一道防线[16]。研究发现，EBV相关地方性Burkitt淋巴瘤（多为原发EBV感染引起）患者体内功能紊乱的CD56^-^CD16^+^ NK细胞亚群的异常增多，而其杀伤能力下降[17]。Wiesmayr等[18]对比了EBV血清学阴性的儿童接受实体器官移植后发生EBV-PTLD和无症状EBV感染NK细胞亚群及功能的差异，发现PTLD患儿的CD56^-^CD16^+^ NK细胞亚群扩增，CD56^bri^ NK细胞亚群杀伤能力下降，总NK细胞群耗竭标志物PD-1表达升高。这些结果提示在EBV原发感染引起的相关疾病中存在NK细胞功能受损现象。NK细胞在EBV潜伏感染激活中的免疫作用研究较少。我们的结果显示单倍体HSCT后1个月，EBV+组CD16^bri^CD56^dim^细胞亚群上CTLA-4的表达高于EBV−组。Juan等[14]对比11例allo-HSCT后EBV+患者和11例EBV−患者，发现HSCT后EBV+患者中CD56^bri^ NK细胞比例更高，且Ki-67表达亦高于EBV−患者，发生EBV-PTLD的患者Ki-67、CD107a、IFN-γ在总NK细胞和CD56^bri^亚群中的表达降低。CD56^bri^亚群扩增、Ki-67高表达被认为有助于EBV感染的控制，而CD56^bri^亚群扩增降低，Ki-67、CD107a、IFN-γ等细胞因子低表达则提示NK细胞控制EBV感染能力可能受损。我们的研究发现移植后2、3个月EBV+患者相较EBV−患者CD16^neg^CD56^bri^亚群中位比例升高及CD16^neg^CD56^dim^耗竭标志物TIM-3的表达下降，同时EBV+患者CD16^bri^细胞亚群表达更多的颗粒酶B和穿孔素。本研究中移植后EBV激活的中位发生时间为+45（+14～+81）d，在移植后2及3个月时大部分EBV激活患者正在感染EBV或已经历过EBV感染，且均未进展为EBV疾病。我们认为NK细胞在EBV刺激下通过释放细胞因子和趋化因子，发挥重要的抗病毒作用，从而在一定程度上控制EBV感染。

T细胞耗竭是病毒免疫逃逸的主要机制之一[6]。实体器官移植患者移植后发生EBV激活时外周血T细胞表达耗竭表型，PD-1表达增高，EBV-CTL抗病毒功能受损[19]–[20]。EBV阳性B细胞淋巴瘤人源化小鼠模型研究显示阻断PD-1可增加IFN-γ和TNF-α，恢复抗病毒功能[21]。Huang等[13]的研究显示allo-HSCT患者移植后30 d CD8^+^和CD4^+^CD45RO^+^比例与EBV激活风险相关。与之相似，我们的结果显示移植后1个月EBV+患者CD3^+^CD8^+^ T细胞比例低于EBV−患者。我们发现EBV+患者CD3^+^CD8^+^和CD3^+^CD4^+^ T细胞TIM-3表达均增高。我们还观察到EBV+患者T细胞颗粒酶B和IFN-γ的表达低于EBV−患者。这些结果提示allo-HSCT后发生EBV激活患者存在T细胞功能受损。

我们的研究样本量较小，扩大样本研究移植后T细胞亚群尤其是EBV-CTL的重建、功能和耗竭分子的表达，可能对预测移植后EBV激活和早期干预具有一定的临床意义。同时，这些免疫细胞亚群在移植后EBV激活中的具体通路机制也值得进一步探讨。

总之，本研究结果提示单倍体HSCT后T淋巴细胞重建延迟、耗竭分子高表达及杀伤功能减弱可能增加EBV激活的风险。这些发现为进一步阐明HSCT后EBV激活的机制提供了理论依据。
